# Faculty Perceptions of Online Teaching Effectiveness and Indicators of Quality

**DOI:** 10.1155/2017/9374189

**Published:** 2017-02-23

**Authors:** Christine Frazer, Debra Henline Sullivan, Deborah Weatherspoon, Leslie Hussey

**Affiliations:** School of Nursing, Walden University, 100 Washington Avenue South, Suite 900, Minneapolis, MN 55401, USA

## Abstract

Online education programs in nursing are increasing rapidly. Faculty need to be competent in their role and possess the skills necessary to positively impact student outcomes. Existing research offers effective teaching strategies for online education; however, there may be some disconnect in the application of these strategies and faculty perceptions of associated outcomes. Focus groups were formed to uncover how nursing faculty in an online program define and describe teaching effectiveness and quality indicators in an asynchronous online environment. A semistructured interview format guided group discussion. Participants (*n* = 11) included nurse educators from an online university with an average of 15 years of experience teaching in nursing academia and 6 years in an online environment. Teaching effectiveness, indicators of quality, and student success were three categories that emerged from the analysis of data. What materialized from the analysis was an overarching concept of a “dance” that occurs in the online environment. Effective online teachers facilitate, connect, lead, and work in synchrony with students to obtain indicators of quality such as student success, student improvement over time, and student application of knowledge to the professional role.

## 1. Introduction

Online education programs in nursing are growing in increased numbers [1, 2]. Online programs range from associate to doctoral degrees. With the growing number of online education programs in nursing, faculty need to be competent and possess skills specific to the online learning environment. Effective teaching strategies foster student learning, satisfaction, and achievement of outcomes [3–5]. A literature review of research on effective online teaching strategies revealed several recurrent themes of collaborative activities such as discussion boards, instructor presence, and using a variety of instructional methods [6]. Plante and Asselin [7] examined online nursing education and found that several factors are important to create a sense of social presence and caring. Richardson et al. [8] also examined presence and supported common factors. These factors include the ability to provide respectful, encouraging, timely, and positive faculty messages while, concurrently, allowing caring interactions between faculty and students, establishing mutual respect, and finding meaning in the faculty-student relationship [7, 8]. However, there may be a disconnect between application of these strategies and faculty perception of the most important elements for student success.

There is a gap in the literature of qualitative studies that focus on nursing faculty's perceptions of teaching effectiveness in an online environment in nursing. An integrative review by Horne and Sandmann [9] stated that more studies are needed that evaluate online programs, especially at the graduate level. One pilot qualitative study used focus groups to explore faculty perceptions of how a workshop supported the ability to implement best practices into their courses and allowed students to provide feedback [10]; a limitation of this study includes that it was a specific evaluation of a workshop and has limited transferability. Carter et al. [11] conducted a mixed methods study in Canada to discover what students and faculty view as strategies that ensures meaningful e-learning. Focus groups elicited answers to questions regarding elements that require dedicated support about educational practices and technology aspects so that the e-learning experience is meaningful. Four themes emerged which were the human connection (student-faculty and student-student), IT support, course design that is specific to e-learning, and institutional infrastructure to support e-learning. The researchers on e-learning experiences involving faculty and students recommend a need for additional research. Other studies have focused on online teaching effectiveness, but these have been in other disciplines such as medical education [12] or focused on student perception of e-learning [13]. In a recent review, Zidan [14] states that there is a need for more qualitative studies to address the gap regarding the effectiveness of online learning.

The purpose of this descriptive qualitative study was to investigate nursing faculty perceptions of what constitutes teaching effectiveness and indicators of quality in an online learning environment and to determine if faculty perceptions align with current best practices for online education.

## 2. Method

To examine instructors' perceptions of teaching effectiveness within the online environment, a focus group approach, as described by Krueger [15], guided the study. Focus group, a method used by social science researchers, is an efficient method of obtaining qualitative data from multiple participants [16]. Focus groups are less threatening and may provide a deeper understanding of the phenomena by encouraging group participants to make comparisons between their experiences and other instructor experience [16]. Comparison, in turn, highlights either consensus or diversity of experiences on a topic [16].

### 2.1. Participants

Purposive sampling sought participants teaching at a university located in the United States known to have experience in nursing academia and online learning environments. Recruitment of participants occurred via an email invitation to participate in a one-hour teleconference. Inclusion criteria were that participants were currently teaching online and had taught at least two years within an online learning environment. The final sample consisted of 11 participants. All participants were doctoral prepared, and the group had an average of 15 years of experience teaching and six years in teaching in an online environment. At the time of the focus group sessions, three participants teach in an online baccalaureate nursing program, and eight teach in an online master's degree in nursing program.

IRB approval was obtained, and adequate provisions to protect the privacy of subjects and to maintain the confidentiality of the data were employed.

### 2.2. Design

In this qualitative, descriptive study, focus group methodology, as described by Krueger and Casey [16], guided data collection and analysis with an aim to uncover online nursing faculty perceptions of what constitutes teaching effectiveness, indicators of quality, and to share personal examples in a nonthreatening group discussion. Unlike one-on-one interviews, focus groups permit participants to express and clarify their views, creating a synergy of information that is valid [17]. Participants were divided into two focus groups, based on participant availability, for a scheduled one-hour group teleconference interview with the principal investigator (moderator) and coinvestigator (assistant moderator). Group sessions were recorded via InterCall, an online conference account management tool, and, as a backup, audio-recorded. Coinvestigator confirmed a connection to InterCall and audio device recording before each focus group session. To assure accuracy, each session was transcribed verbatim and accuracy verified by the investigators.

A semistructured interview format guided the discussions [16]. As moderator, the principal investigator facilitated the discussion. The interview began with a series of questions regarding participants' current title and position, years of experience in academia and an online environment, and, lastly, the academic level taught in nursing. In each focus group, participants responded to the following questions:

- How would you define effective online practices in teaching?
- What are some examples of effective online teaching practices?
- What impact does an effective teaching practice have on students?
- How do you define quality in an online environment?
- What indicators of quality have you observed and utilized in an online environment?
- What impact do indicators of quality have on students?

Before ending each focus group session, the principal investigator summarized the main points of the feedback and asked participants if they had any questions, clarifications, or additional comments they would like to share. After additional comments and questions, both investigators thanked participants for their participation, and the session ended. In total, each session lasted 80 minutes. Focus groups that are well designed usually last between 1 and 2 hours [15].

### 2.3. Data Analysis

Constant comparison analysis [18, 19], a data analysis technique first used in grounded theory, can also be used to examine focus group data [20]. In this study, researchers employed this method of analysis. First, each researcher analyzed data line by line based upon the audiotape transcripts. Analyzing data line by line is considered the most rigorous [15, 21]. During line-by-line analysis, codes were given to words or phrases that represented units of data associated with an idea. For example, words or phrases associated with quality were coded “RQ” for representing quality. If additional clarity was needed from a participant after reviewing transcripts, the lead researcher directly contacted participant. In total, the lead researcher contacted only two participants for additional clarity in regard to their comments. Then, working together, the researchers grouped codes into categories that best fit the data. The categories that were apparent related directly to the questions asked in the focus groups:* teaching effectiveness* and* indicators of quality*. However, another category that was not one of the questions emerged as* student success*. Lastly, in the final stage of constant comparison analysis, one or more themes are developed to express content of each of the grouped categories [18, 19]. Through a sequence of investigative sessions among researchers, what materialized to express content of each of the categories was an overarching concept of a “dance” that occurs in the online environment.

## 3. Findings

To present a meaningful and well-defined picture, presented first are findings associated with each category. Subsequently, depicted is the relevance visualized by researchers through the concept of a dance within the online environment.

### 3.1. Teaching Effectiveness

The participants' extensive experiences provided rich descriptions that helped the researcher to understand their perceptions of online effective teaching strategies. The participants viewed effective online teaching practices as an instructor who (a) facilitates student learning, (b) aims to feel connected with students in the classroom, (c) shares experiences, (d) is approachable, (e) establishes mutual comfort, and (f) is responsive to students' needs. The following excerpts from the transcripts support these findings:

I really see myself more of a facilitator when I am in the online environment, rather than a teacher … I want students to feel comfortable… to feel connected in the online environment…I try to share real life stories and also try to build on their experiences…I want them to know they can reach out and ask questions… Some students like one-on-one, some of them like that conference call, others only want to do email; it just depends on the type of student…you need to offer different mechanisms to communicate to be responsive to their needs.

Participants also expressed the importance of stimulating students to explore new thought, keeping them challenged and leading students to the spotlight during the discussion and, in turn, having those invite others into a scholarly dialog:

I ask a question about what a student said in their discussion post …did it stimulate their thought… did it stimulate somebody else's thought…did it stimulate more questions rather than just being a pat on the head?

Additionally, agreement on the notion of “keeping the brain wheels turning” was viewed as teaching effectively to lead students:

…I think if you can keep them thinking instead of just feeling they are writing and doing an assignment, it sparks their learning. Once you send them a question and they answer you back, you can see that you made the wheels keep turning.

In both focus groups, participants expressed that effective online teaching practices play a role in reducing student stress, improving student work, critical thinking, and their receptiveness to feedback. In regard to critical thinking, participants linked student improvement and critical thinking to instructor feedback and questioning. For example, participants commented with the following:

Instructors who are evaluating written work…with very specific feedback…students in the next assignment improve in an area of their written work…when instructors then ask questions that make the students think more deeply about a topic in the discussions, they [students] actually are finding out new information or delving into the topic more deeply than they would have otherwise without the instructor's guidance…

In addition to instructor feedback to improve student learning and critical thinking, adjustments based on student feedback to instructor was deemed as an essential part of effective online teaching that impacts student outcomes:

I actually enjoy receiving feedback from students …they are able to say what you did really helped me to learn…for example, they appreciate the conference call with the class…and they would like to have that happen again…I also look at the feedback in great detail to see what it is that I can do to improve my teaching online…is what I am doing meeting students' individual needs… making those adjustments as needed…

### 3.2. Student Success

An indicator of achievement perceived was student success. Participants viewed success as students successfully passing the course, demonstrating improvement, and practicing the learning. One participant stated, “if you have a class of 30 and only 10 passed the course successfully, then that is a clue…something is wrong….” Another participant added, “I had maybe three students who really did improve over time, and they passed, so I had 100 percent pass rate, but really, the quality indicator was in the improvement over time.” Overall, focus group participants voiced that although students can meet course objectives, improve over time, and pass the course, the essential factor associated with success was the student's ability to apply knowledge gained within their professional role:

I think we have to look at our graduates and say, was what we taught you what you needed to know to succeed in the real world beyond just whether you passed…if they don't have the skills they need to survive when they get out and work in our discipline, it's not going to be pretty…students are not in school to get that master's degree, just that piece of paper, but to apply new skills and knowledge to improving their overall job…or their future plans…

### 3.3. Indicators of Quality

Additionally, focus group participants perceived effective teaching strategies were successful when instructional quality indicators, as defined and measured by the organization where they were employed, are achieved. Such indicators included time spent within the online environment, the number of days posted within the online environment, the number of responses made to students within a discussion, and timeliness of responses to students' questions and grading of assignments. In essence, participants viewed organizational measures of quality associated with instructor presence: “being present in the classroom at least four times a week…responding to at least two-thirds of the class on a personal basis…and get input back to students within seven business days….” However, participants agreed that when it came to participating in online discussions or counting the number of days present within the classroom, it was not the number of posts or days that mattered most, it was what instructors do when they are in the online classroom. As for discussion boards, participants felt although they wanted to engage actively, it was a “balancing act” regarding how often the instructor should intervene:

I have one class that is just taking off. They have their own probing questions that take the discussion to a new level. I don't need to do that, you know, they're just phenomenal…you don't want to be in there too much because you can inhibit the discussion…you don't want to interfere with the student sense of community and collaborative learning either.

### 3.4. The Concept of Dance within the Online Environment

As the categories emerged, connections in the form of interactions between instructor, student, and content were apparent. The concept of a “dance” represents the process that best describes the fluid nature of what constitutes good teaching practices in an online environment. The theme embodies the recurring dialog evidenced in the data that sometimes the instructor needs to lead and sometimes they need to follow, allowing the student to lead. The dialog includes allowing students to interact freely with one another in order create an environment where students feel comfortable sharing viewpoints and personal experiences. The dance becomes more diverse and interesting as the student exchanges partners for a student-student relationship bringing additional experiences and opinions to the topic of discussion. It also includes the interaction and movement between content and application of experiences creating synergy and development of critical thinking skills. Furthermore, visualizing teaching effectiveness as a choreographed dance performed on the stage of the online classroom (i.e., online platform) exemplifies existing best practices for online education including presence, interaction, respect, encouragement, and timely interaction.

Student success was another category that emerged from the focus groups. Within the concept of dance that occurs in the online environment, the researchers visualized students as “future stars.” When the synergy of the student-faculty and student-student dance improves, transpiring of successful learning begins and the spotlight on the dance performance stage (online platform/online learning environment) illuminates brighter.

The last category formed was indicators of quality. This category represents measurable components of success. As the dance continues between instructor-students and student-students, an overall artistic vision emerged as a “standing ovation.” This is interpreted as the allegory for an achievement of tangible quality indicators/outcomes of the dance (learning process) performance for the student (stars) and instructor.

## 4. Discussion

Nursing has adapted to the changing educational environment by including the online format in the delivery of nursing education. Initial skepticism gave way to questioning the quality and effectiveness of this popular way of educating future nurses [2]. The demand for this format propagates the need for an investigation into best practices to assure no compromise of the outcome of producing competent nurses. Online education in various forms has existed within the US traditional higher education system for some time and continues to grow due to the demand [1]. Benchmarks set by the American Online Education Consortium (ADEC), the American Federation of Teachers, National Education Association (NEA), and the Quality Matters project all suggest the road to assuring quality and the commonalities of active learning, timeliness of feedback, level of interaction, and applicable instructional materials are important in the online course design [22]. In the present study, participants expressed, as in the benchmarks, the importance of active learning and timely feedback with a special emphasis on personal interaction. The focus groups data led to this personal interaction as the central focus for high teaching effectiveness with quality outcomes to occur.

Since the 1990s, the paradigm shifted from instruction to learning as a collaborative process with learning outcomes as described by Barr and Tagg [23] and applications are most evident in online education. The result is a shift in the role of the instructor to a skilled facilitator and partner in the production of learning [24, 25].

Chickering and Gamson's [26] seven principles provide a guide for supporting online nursing education. These seven principles include encouraging student-teacher contact, cooperation among students, active learning, providing prompt feedback, emphasizing time on task, conveying high expectations, and respect for diverse talents and ways of learning [26]. Researchers have utilized these principles as an approach to creating, implementing, and evaluating an online course. In Crews et al.'s [27] study, researchers applied Chickering and Gamson's principles to online course design to enhance student success. Dusaj [28] examined Chickering and Gamson's seven principles and presented a variety of instructional strategies, representative of best practices in online nursing courses, which would fall under each of these principles. Several of the instructional strategies portrayed the importance of connecting, adapting, directing, and feedback that needs to occur in the online platform between the instructor and student. Edwards et al. [29] found that students identify exemplary face-to-face and online instructors as those that are challengers, affirmers, and influencers while creating presence in the classroom. Although principles infer effective communication, they do not specifically address the simultaneous movement of the relationship needed in the online environment. These studies do support similar expectations of both instructor and learner as they come together in an online classroom [26–29].

An experience that includes the interaction with a problem and others discussing the same problem continues to emerge as a basis for learning, particularly for online learning. Kolb's [30] Experiential Learning Theory (ELT) explains the cyclical process learners use to move beyond data memorization, or cognitive gain, and into critical thinking to support decision-making [31]. This active learning process takes place during real or simulated experiences. In the online environment, the use of interactive discussion provides a venue for online educators to engage in the ELT learning cycle where the simultaneous movement of the relationship is clearly explained [30].

Kolb's [30] theory posits that learning begins with a problem (the discussion question) followed by the learner's critical thinking or reflecting on personal knowledge and experience. Questioning provides students with the opportunity to consider course learning resources applied to the discussion problem and simultaneously bring personal experience into the discussion dialog. Learning continues as the instructor, or another student, stimulates deeper thinking with a well-formed question or comment and spurs the student to assert their position on the issue. While at times the instructor leads the dialog with a challenging question, often a small prompt allows the student to take the lead and support their decision (discussion posting). This action, or the stand that the student is encouraged to verbalize and explain, leads the student to examine further his or her understanding and decision-making. Quality online instruction requires instructors to accurately assess when a student needs to be led or simply needs to know that a partner is following them.

Garrison's Community of Inquiry (CoI) model [32] appropriately lends itself to the analysis of the instructor-student interaction because the framework addresses the social, cognitive, and teaching presence as concentric circles that overlap and create synchronous movement within the interaction. Study participants viewed the interaction that takes place in the online learning environment as the essence of teaching effectiveness and quality indicators that enhance positive outcomes. In the CoI model, social presence (SP) explores the feeling of connectedness to one another. Carter et al. [11] support the importance of connectedness which is the center of a meaningful online learning experience. Cognitive presence (CP) focuses on the process in which students build and confirm meaning. Lastly, teaching presence (TP) centers on instructional methods utilized to enhance quality and potential outcomes in the online environment. Previous research identified TP as the community pillar [4] and meaningful learning outcomes [11]. The findings from this study also suggest TP, the teaching effectiveness in the online environment, is an important factor in building SP and CP. Instructional methods such as sharing experiences, communicating through announcements, phone calls, and emails, answering questions, providing detailed feedback, and asking probing/prompting questions in the discussion forums lend themselves not only to building that feeling of connection in an online environment but towards student reflection and construction of meaning.

## 5. Limitations

There are several limitations to this study. Respondents volunteered and were purposively selected from one online university. Therefore, data produced from this study may not fully represent the general population of nursing faculty who teach. Future recommendations would include participants from a variety of online universities and various programs of study.

The relationship between the researcher and the participant is one that has potential for exploitation of study participants if not carefully monitored [33]. In this case, the researchers who led the focus groups were faculty at the same institution as the participants, which could have resulted in some bias. Researchers are obligated to anticipate and recognize the potential impact the leader of a focus group may have on subjects and minimize associated risks as much as is possible, and this was done [33].

## 6. Conclusion

In conclusion, this study adds to the body of evidence supporting best teaching practices for online instruction. The literature revealed effective online teaching strategies with several recurrent themes of collaborative activities such as discussion boards, instructor presence, and using a variety of instructional methods [6]. In addition, findings support that instructors' perception of best practices and quality outcomes align with current literature. For example, Plante and Asselin [7] and Richardson et al. [8] examined online nursing education and found that several factors are important to create a sense of social presence and caring by providing respectful, encouraging, timely, and positive faculty messages while, concurrently, allowing caring interactions between faculty and students, establishing mutual respect, and finding meaning in the faculty-student relationship. The participants' perceptions aligned with these strategies of creating a social presence and caring as they viewed effective online teaching practices as an instructor who (a) facilitates student learning; (b) aims to feel connected with students in the classroom; (c) shares experiences; (d) is approachable; (e) establishes mutual comfort; and (f) is responsive to students' needs.

The number of online education programs in nursing will continue to grow and expand. To accommodate this expansion and growth, there will be a need for instructors who demonstrate teaching effectiveness in an online environment. Several suggestions to enhance instructor knowledge of teaching effectiveness are receiving guidance by faculty mentors, feedback from student and peer evaluations, sharing of best practices among faculty in established e-college (online) communities or forums, and orientation programs for instructors transitioning into an online role.
